# Single-Isocenter Volumetric Modulated Arc Therapy vs. CyberKnife M6 for the Stereotactic Radiosurgery of Multiple Brain Metastases

**DOI:** 10.3389/fonc.2020.00568

**Published:** 2020-05-08

**Authors:** Rami A. El Shafie, Eric Tonndorf-Martini, Daniela Schmitt, Aylin Celik, Dorothea Weber, Kristin Lang, Laila König, Simon Höne, Tobias Forster, Bastian von Nettelbladt, Sebastian Adeberg, Jürgen Debus, Stefan Rieken, Denise Bernhardt

**Affiliations:** ^1^Department of Radiation Oncology, Heidelberg University Hospital, Heidelberg, Germany; ^2^National Center for Radiation Oncology (NCRO), Heidelberg Institute for Radiation Oncology (HIRO), Heidelberg, Germany; ^3^Institute of Medical Biometry and Informatics (IMBI), Heidelberg University Hospital, Heidelberg, Germany; ^4^Clinical Cooperation Unit Radiation Oncology (E050), German Cancer Research Center (DKFZ), Heidelberg, Germany; ^5^Deutsches Konsortium für Translationale Krebsforschung (DKTK), Partner Site Heidelberg, German Cancer Research Center (DKFZ), Heidelberg, Germany; ^6^Department of Radiation Oncology, University Medical Center Göttingen, Göttingen, Germany

**Keywords:** multiple brain metastases, radiosurgery, palliative, radiotherapy, whole-brain radiotherapy, stereotactic, linear accelerator, robotic radiosurgery

## Abstract

**Introduction:** Stereotactic radiosurgery (SRS) is becoming more frequently used for patients with multiple brain metastases (BMs). Single-isocenter volumetric modulated arc therapy (SI-VMAT) is an emerging alternative to dedicated systems such as CyberKnife (CK). We present a dosimetric comparison between CyberKnife M6 and SI-VMAT, planned at RayStation V8B, for the simultaneous SRS of five or more BM.

**Patients and Methods:** Twenty treatment plans of CK-based single-session SRS to ≥5 brain metastases were replanned using SI-VMAT for delivery at an Elekta VersaHD linear accelerator. Prescription dose was 20 or 18 Gy, conformally enclosing at least 98% of the total planning target volume (PTV), with PTV margin-width adapted to the respective SRS technique. Comparatively analyzed quality metrics included dose distribution to the healthy brain (HB), including different isodose volumes, conformity, and gradient indices. Estimated treatment time was also compared.

**Results:** Median HB isodose volumes for 3, 5, 8, 10, and 12 Gy were consistently smaller for CK-SRS compared to SI-VMAT (*p* < 0.001). Dose falloff outside the target volume, as expressed by the gradient indices GI_high and GI_low, was consistently steeper for CK-SRS compared to SI-VMAT (*p* < 0.001). CK-SRS achieved a median GI_high of 3.1 [interquartile range (IQR), 2.9–1.3] vs. 5.0 (IQR 4.3–5.5) for SI-VMAT (*p* < 0.001). For GI_low, the results were 3.0 (IQR, 2.9–3.1) for CK-SRS vs. 5.6 (IQR, 4.3–5.5) for SI-VMAT (*p* < 0.001). The median conformity index (CI) was 1.2 (IQR, 1.1–1.2) for CK-SRS vs. 1.5 (IQR, 1.4–1.7) for SI-VMAT (*p* < 0.001). Estimated treatment time was shorter for SI-VMAT, yielding a median of 13.7 min (IQR, 13.5–14.0) compared to 130 min (IQR, 114.5–154.5) for CK-SRS (*p* < 0.001).

**Conclusion:** SI-VMAT offers enhanced treatment efficiency in cases with multiple BM, as compared to CyberKnife, but requires compromise regarding conformity and integral dose to the healthy brain. Additionally, delivery at a conventional linear accelerator (linac) may require a larger PTV margin to account for delivery and setup errors. Further evaluations are warranted to determine whether the detected dosimetric differences are clinically relevant. SI-VMAT could be a reasonable alternative to a dedicated radiosurgery system for selected patients with multiple BM.

## Introduction

For patients with up to three or four brain metastases, stereotactic radiosurgery (SRS) is the treatment of choice, according to current guidelines (1, 2). However, recent data have suggested that patients with multiple—meaning up to 10—metastases may benefit equally from SRS: In a large prospective observational trial, Yamamoto et al. showed overall survival following SRS to be similar between patients with 2–4 lesions, compared to patients with 4–10 lesions (3). Several phase 3 trials have shown SRS to be significantly less toxic than whole-brain irradiation (WBRT), the hitherto established treatment for multiple brain metastases (4–6).

Until recently, the feasibility of SRS for multiple brain metastases was limited by technical reasons: with the exception of the dedicated radiosurgery systems Gamma Knife (Elekta Stockholm, Sweden) and CyberKnife (Accuray Inc. Sunnyvale, California), the respective workflow at a conventional linear accelerator (linac) was cumbersome and time consuming. In general, a separate isocenter would be required for every treated lesion, which requires repeated position verification during treatment delivery. Additionally, treatment using sequential multifield 3D-conformal radiotherapy (3DCRT) or dynamic conformal arc therapy (DCAT) would lead to a significantly increased integral dose to the healthy brain (HB) (7, 8).

Meeting the increased clinical demand and due to the limited availability of the Gamma Knife and CyberKnife systems, several dedicated tools for the SRS of multiple brain metastases at a conventional linac have recently emerged. Most prominent among those are HyperArc™ (Varian, Palo Alto, CA, USA) and Elements™ Multiple Brain Mets (MBM) software by Brainlab (Munich, Germany) (9–11). Those tools rely on the use of volumetric-modulated arc therapy (VMAT) or DCAT to treat multiple lesions with a reduced set of isocenters or a single isocenter, featuring varying degrees of automation at the treatment planning level. Limited data have suggested comparability of those techniques to dedicated radiosurgery systems in terms of dose conformality, while potentially achieving a reduction in treatment time (10, 12). However, the few reports available on this subject focus on patients with limited (usually <5) metastases and rather larger volumes per lesion.

RaySearch's RayStation V8B (RaySearch, Stockholm, Sweden) provides the technical requirements for creating single-isocenter VMAT plans similarly to the abovementioned dedicated tools, while providing a degree of automation with the use of adaptable plan templates and the comprehensive scripting capabilities. However, a systematic dosimetric comparison with a dedicated radiosurgery system in this regard has not yet been done.

In the current work, we compared single-isocenter VMAT, planned on RayStation V8B for delivery at an Elekta Versa HD linac (Elekta, Stockholm, Sweden) with the CyberKnife M6 system for the SRS of multiple (5–10) brain metastases. Systematic comparison was done regarding different plan quality metrics, such as conformity and gradient indices, integral dose to the HB and treatment time.

## Patients and Methods

We identified 20 treatment plans for patients who received CyberKnife-based single-session SRS (CK-SRS) for five or more brain metastases at our institution in 2017 and 2018. For every CyberKnife treatment plan, we calculated an alternative plan using single-isocenter VMAT according to the specifications outlined below.

All analyses were performed following institutional guidelines and the Declaration of Helsinki of 1975 in its most recent version. Ethics approval for the study and a waiver of written informed consent was granted by the Heidelberg University ethics committee on April 12, 2018 (#S-172/2018). Patient confidentiality was maintained by anonymizing patient data to remove any identifying information.

### Imaging and Target Definition

Treatment planning for all patients was based on high-resolution computed tomography (CT) and magnetic resonance imaging (MRI). During CT scan and treatment, patients were fixated with the help of an individually fitted thermoplastic mask. Standardized imaging protocols were used for all patients, complying to the following specifications: CT scan was acquired with 1-mm slice thickness. MRI contained a contrast-enhanced, T1-weighted, three-dimensional sequence with multiplanar reconstruction and a slice thickness of ≤1 mm. The MRI was thoroughly coregistered and served as basis for target and organs at risk (OAR) delineation. Gross tumor volume (GTV) consisted of all contrasted tissue in the T1-weighted MRI. Considering the availability of intrafractional tracking and motion compensation, a safety margin of 1 mm was added to the GTV by isotropic expansion to create the planning target volume (PTV) for CyberKnife treatment. The same approach was applied, expanding the GTV by 3 mm to create the PTV for SI-VMAT for delivery at the Elekta Versa HD linac installed at our facility, where no such intrafractional motion compensation is currently available. The choice of respective margin width was done following literature recommendations and established institutional practice (8, 13, 14).

### Treatment Planning

Dose prescription was done according to metastasis size and in compliance with current guidelines (15, 16). Prescribed margin doses were 20 or 18 Gy to the 70% isodose, covering at least 98% of the PTV with the objective of achieving best possible conformity for the sum of all PTVs. Dose constraints for OAR, specifically brain stem and optical tract were observed according to QUANTEC data and literature recommendations (15, 17, 18). All treatment plans were reviewed for clinical acceptability by a radiation oncologist experienced in intracranial SRS.

Treatment planning for CyberKnife was performed in Accuray's Multiplan v5.3 and subsequent versions (henceforth referred to as “CyberKnife TPS”), while treatment was delivered using CyberKnife M6 (Accuray Inc., Sunnyvale, CA, USA). Using sequential optimization, the region of 10% isodose was held as low as possible with the objective of no 20% dose regions outside the direct target vicinity. For targets close together, tuning structures to split the high-dose regions between the targets were also used. Treatment time was reduced during the optimization through multiple “beam reduction” steps until plan quality degraded too much, meaning mainly a reduced coverage below 98% for any single PTV or the appearance of dose regions with 20% (or higher) outside the direct target vicinity.

**Figure 1:**
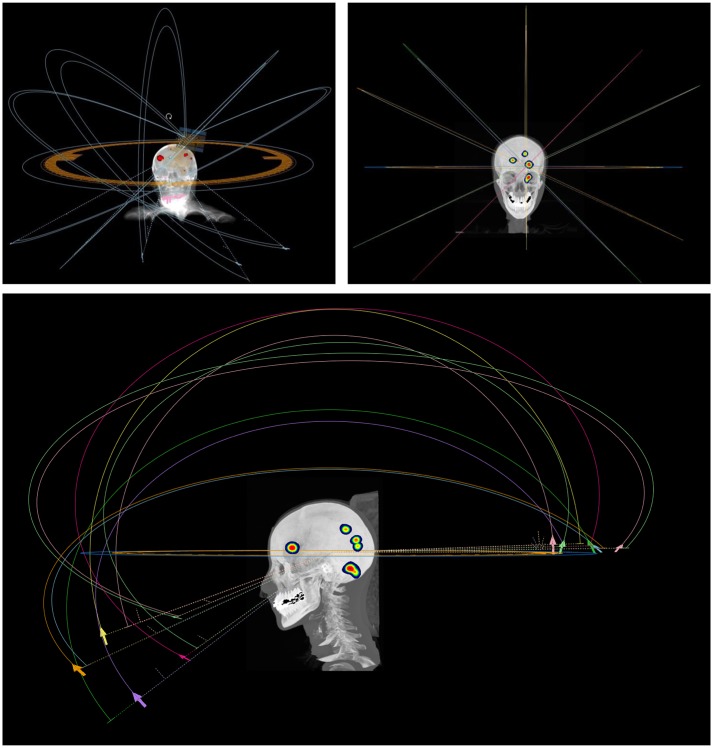
Arc arrangement for single-isocenter volumetric modulated arc therapy (SI-VMAT); treatment planning was based on the use of six non-coplanar dual arcs with fixed couch angles of 0, 25, 45, 90, 315, and 335°.

Treatment planning for SI-VMAT was performed on RaySearch's RayStation V8B, utilizing the comprehensive scripting capabilities for semiautomated plan generation and analysis. The single isocenter was positioned at the centroid of the sum of all target lesions. Plan design was based on the use of six non-coplanar dual arcs with fixed couch angles of 0, 25, 45, 90, 315, and 335° (Figure 1). Treatment plans were generated for delivery at a VersaHD™ linear accelerator (Elekta, Stockholm, Sweden) with an Agility™ (Elekta Stockholm, Sweden) multileaf collimator (MLC) featuring 5 mm leaves at the isocenter and flattening filter-free (FFF) application. Dose distributions were computed, with 1 mm of dose-grid step and 4° of angular step (control point) along the arcs. The algorithms used for dose calculation were Ray Trace for CyberKnife and Collapsed Cone for SI-VMAT.

### Comparison Metrics

Dose distributions were exported and processed with the DVHmetrics package for R for analysis to ensure fair and independent computation of dose statistics (19). The HB contour was created for every treatment plan by subtracting the sum of all PTVs (PTV_all) from the whole brain contour. The healthy brain volumes receiving 12, 10, 8, 5, and 3 Gy (*V*_12Gy_, *V*_10Gy_, *V*_8Gy_, *V*_5Gy_, and *V*_3Gy_) were determined and compared between SI-VMAT and CK-SRS.

Dose conformity to the PTV was assessed using the Radiation Therapy Oncology Group (RTOG) conformity index (CI) and the new conformity index (nCI). The CI is defined as the quotient of the prescription dose volume (*V*_pi_) and the target volume (*V*_PTV_), as follows (20):

$$\begin{array}{l} \text{CI}=\frac{V_{\text{Pi}}}{V_{\text{PTV}}} \end{array}$$

The nCI is defined as the reciprocal of the modified Paddick conformity index as follows (21, 22):

$$\begin{array}{l} \text{nCI}=\frac{V_{\text{PTV}}\times \;V_{\text{pi}}}{{\left(V_{\text{PTV},\text{ pi}}\right)}^{2}} \end{array}$$

Herein, *V*_PTV_ is the planning target volume, *V*_pi_ is the body volume of the patient covered by the prescribed dose, and *V*_PTV, pi_ is the partial volume of the PTV covered by the prescribed dose. The nCI measures the quality of target coverage while considering the dose spilled to healthy tissue outside the target volume. CI and nCI are commonly employed quality parameters in radiosurgery and take a value of 1 for ideal conformity, while values above 1 denote less conformal treatment plans.

Two gradient indices, as described by Paddick et al. and modified by Stieler et al. were calculated to assess dose falloff outside the target volume (23, 24):

$$\begin{array}{l} {\text{GI}}_{\text{high}}=\;{\text{V}}_{50\text{\%Presc}.\text{Dose}}/{\text{V}}_{90\text{\%Prescr}.\text{Dose}} \end{array}$$

and

$$\begin{array}{l} {\text{GI}}_{\text{low}}=\;{\text{V}}_{25\text{\%Presc}.\text{Dose}}/{\text{V}}_{50\text{\%Prescr}.\text{Dose}} \end{array}$$

Treatment time for CK-SRS is estimated by the CyberKnife TPS with an underlying kV-fluoroscopy imaging frequency set at one image per minute. An estimate of 5 min for patient setup and initial alignment is furthermore included in the calculation. In clinical reality, the imaging interval is initially set at 20 s and adjusted according to the trend of patient motion up to a maximum interval of 90 s. In our experience, therefore, the estimate provided by CyberKnife TPS is fairly accurate. For VMAT plans, beam-on time was calculated by RayStation from the number of monitor units (MUs), at an underlying variable dose rate of 500–1,400 MU/min for 6-MV flattening filter-free (FFF) irradiation. An additional 5 min were added to beam-on time to account for patient setup and position verification via kV-ConeBeam CT (CBCT), in accordance with data previously published for our institution (8).

### Statistical Analysis

Descriptive statistics are calculated for baseline characteristics and dosimetric parameters; continuous variables are given as median [interquartile range (IQR) and range] and/or means (SD) and categorical variables as absolute and relative frequencies. Regarding dosimetric analyses, normality assumption may be violated due to the small sample size (*n* = 20), and therefore, non-parametric statistical methods were used. Both treatment plans were developed for each patient, which leads to paired data. All method comparisons were done using the Wilcoxon signed-rank test for paired data. Since this was primarily an experimental analysis, *p*-values are attributed no confirmatory character. An α-level of 5% was used; however, due to their exploratory nature, analyses are not adjusted for multiple testing. Statistical analyses were performed with the software *R version 3.5.1*.

## Results

Baseline characteristics are displayed in Table 1. Median age at the time of SRS was 60 years (IQR, 52–68). The median number of lesions per patient was 6 (IQR, 5–7); 123 lesions were treated in total. The median metastasis volume was 0.07 cc (IQR, 0.02–0.34), and the median total metastasis volume per patient was 2.4 cc (IQR, 1.4–3.7).

**Table 1:** Baseline characteristics.

**Age (years)**	
Mean	60.4
SD	11.2
Median	60
Q1–Q3	52–68
Min.–max.	41–84
**Number of lesions per patient (*n* = 20)**	
Mean	6.2
SD	1.6
Median	6
Q1–Q3	5–7
Min.–max.	5–10
**Total metastasis volume per patient (ml, *n* = 20)**	
Mean	3.6
SD	4.4
Median	2.4
Q1–Q3	1.4–3.7
Min.–max.	0.4–20.1
**Single metastasis volume (ml, *n* = 123)**	
Mean	0.33
SD	0.78
Median	0.07
Q1–Q3	0.02–0.34
Min.–max.	0.01–7.32
**Prescribed dose (Gy) per metastasis (*n* = 123)**	
20 Gy	121 (98.4%)
18 Gy	2 (1.6%)

**Figure 2:**
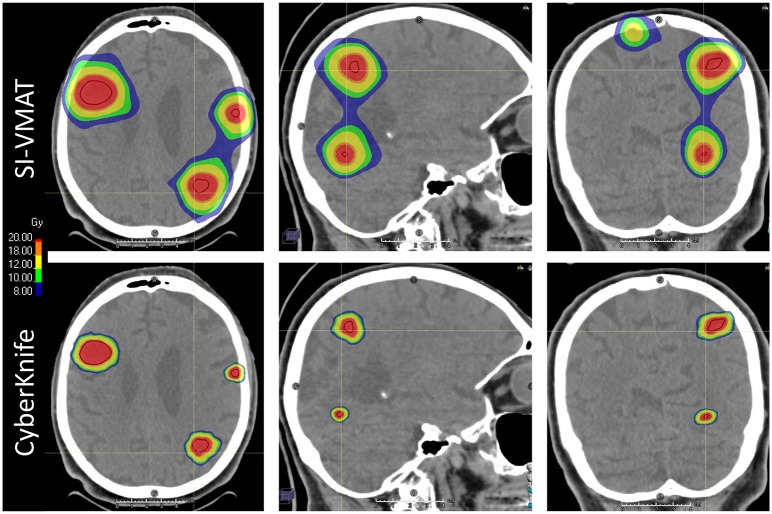
Multiplanar visualization of dose distribution compared between CyberKnife radiosurgery and single-isocenter volumetric modulated arc therapy (SI-VMAT) for a representative case; gross tumor volume (GTV) delineated in dark red. SI-VMAT, single-isocenter volumetric modulated arc therapy.

**Figure 3:**
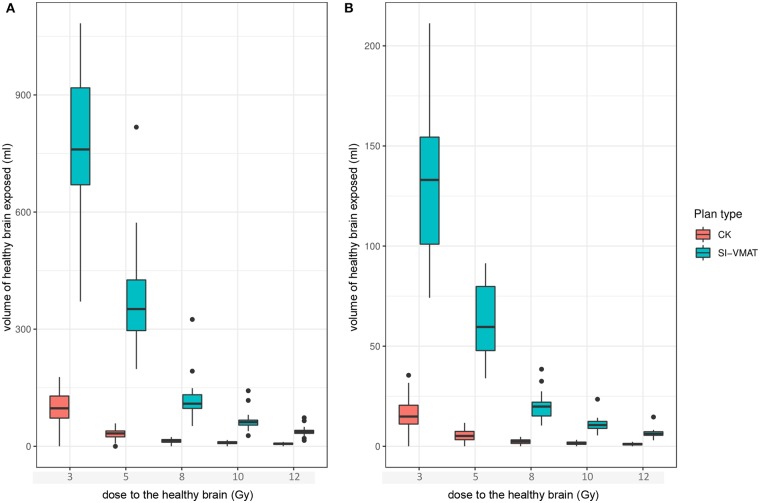
Dose exposure of the healthy brain for different dose levels between 3 and 12 Gy to represent dose falloff for all targets within one treatment plan **(A)** or for the individual target **(B)**; boxes represent Q1–Q3 around the median. CK, CyberKnife; SI-VMAT, single-isocenter volumetric modulated arc therapy; *V*_*X*__Gy_, volume receiving *X* Gy.

All treatment plans in the current analysis fulfilled the criteria for clinical acceptability. Detailed results for dosimetric comparison are displayed in Table 2. Multiplanar dose distribution compared between CK-SRS and SI-VMAT for a representative case is displayed in Figure 2. Median values for the HB volume receiving 3, 5, 8, 10, and 12 Gy (*V*_*X*__Gy_ values) were consistently smaller for CK-SRS compared to SI-VMAT (*p* < 0.001). The differences in isodose volumes for the HB were larger in the low-dose range and decreased in the high-dose range. To quantify dose falloff outside the individual target, the *V*_*X*__Gy_ values for each treatment plan were divided by the number of targets, as suggested by Ruggieri et al. (25). The median *V*_12GyTarget_ thus calculated was 1.1 ml (IQR, 0.6–1.5) for CK-SRS and 6.2 ml (IQR, 5.4–7.3) for SI-VMAT (*p* < 0.001). Results for the dose exposure of the HB are illustrated in Figure 3.

**Table 2:** Dosimetric and treatment parameters in comparison between CyberKnife and single-isocenter volumetric modulated arc therapy (SI-VMAT).

	**CyberKnife**	**SI-VMAT**	***p* (Wilcoxon SR)**
	**Median (Q1–Q3)**	**Median (Q1–Q3)**	
**Dose to the healthy brain**			
Mean dose (Gy)	0.123 (0.109–0.154)	0.413 (0.359–0.445)	**<0.001**
*V*_3GyPlan_ (ml)	97.3 (68.8–128.9)	760.0 (665.2–922.1)	**<0.001**
*V*_5GyPlan_ (ml)	33.0 (23.8–39.6)	351.5 (294.1–429.7)	**<0.001**
*V*_8GyPlan_ (ml)	6.1 (10.1–17.4)	109.1 (95.4–133.8)	**<0.001**
*V*_10GyPlan_ (ml)	9.5 (6.7–12.0)	61.8 (54.1–67.4)	**<0.001**
*V*_12GyPlan_ (ml)	6.5 (4.6–8.3)	37.0 (32.6–41.0)	**<0.001**
*V*_3GyTarget_ (ml)	14.9 (10.5–21.1)	133.0 (100.9–154.5)	**<0.001**
*V*_5GyTarget_ (ml)	5.2 (3.1–7.6)	59.6 (47.5–80.5)	**<0.001**
*V*_8GyTarget_ (ml)	2.4 (1.3–3.3)	19.8 (15.1–22.1)	**<0.001**
*V*_10GyTarget_ (ml)	1.6 (0.9–2.2)	10.6 (8.9–12.5)	**<0.001**
*V*_12GyTarget_ (ml)	1.1 (0.6–1.5)	6.2 (5.4–7.3)	**<0.001**
**Gradients and conformity**			
GI_high_	3.1 (2.9–3.1)	5.0 (4.3–5.5)	**<0.001**
GI_low_	3.0 (2.9–3.2)	5.6 (5.0–6.5)	**<0.001**
CI	1.2 (1.1–1.2)	1.5 (1.4–1.7)	**<0.001**
nCI	1.2 (1.2–1.3)	1.6 (1.5–1.8)	**<0.001**
**Treatment time**			
Estimated treatment time (minutes)	130 (114.5–154.5)	13.7 (13.5–14.0)	**<0.001**

*Numbers in bold indicate statistical significance*.

*Wilcoxon SR, Wilcoxon signed-rank test; V_XGy_, volume of healthy brain receiving X Gy; GI_high_, gradient index for high-dose range; GI_low_, gradient index for low-dose range; CI, RTOG conformity index; nCI, new conformity index*.

Dose gradient outside the target volume, as expressed by the GI_high_ and GI_low_ metrics, was consistently steeper for CK-SRS compared to SI-VMAT. The dose gradient difference between the compared treatment techniques was smaller in the low-dose range than it was in the high-dose range. CK-SRS achieved a median GI_high_ of 3.1 (IQR, 2.9–1.3) vs. 5.0 (IQR, 4.3–5.5) for SI-VMAT (*p* < 0.001). For GI_low_, the results were 3.0 (IQR, 2.9–3.1) for CK-SRS vs. 5.6 (IQR, 4.3–5.5) for SI-VMAT (*p* < 0.001).

Dose conformity was higher for CK-SRS compared to SI-VMAT, as expressed in the consistent difference in CI and nCI values. The median CI for the sum of all PTVs was 1.2 (IQR, 1.1–1.2) for CK-SRS vs. 1.5 (IQR, 1.4–1.7) for SI-VMAT (*p* < 0.001). Respective results for median nCI were 1.2 (IQR, 1.2–1.3) vs. 1.6 (IQR, 1.5–1.8) (*p* < 0.001). Estimated treatment time was relevantly shorter for SI-VMAT, yielding a median of 13.7 min (IQR, 13.5–14.0), compared to 130 min (114.5–154.5) for CK-SRS (*p* < 0.001).

To better assess the impact of the larger PTV in the SI-VMAT treatment plans, a separate comparison has been done for a representative sample of five patients with five to nine BM. For those patients, a second SI-VMAT plan was calculated using the same 1 mm PTV margin also used for CK-SRS treatment plans. Detailed results of this comparison are displayed in Table 3. Qualitatively, the dosimetric results of the comparison of CK-SRS vs. SI-VMAT (1 mm margin) did not differ from the results of the comparison of CK-SRS vs. SI-VMAT (3 mm margin): Median values for the HB volume receiving 3, 5, 8, 10, and 12 Gy (*V*_*X*__Gy_ values) were consistently smaller for CK-SRS compared to SI-VMAT (*p* < 0.001). The same was true for conformity and gradient indices that consistently yielded better results for CK-SRS compared to SI-VMAT. Comparing SI-VMAT (1 mm margin) vs. SI-VMAT (3 mm margin), a minimal decrease in the *V*_*X*__Gy_ values of the HB could be observed for the smaller margin, although the difference did not reach statistical significance for any of the analyzed dose levels. Conformity and gradient indices were worse with the use of a 1-mm margin compared to a 3-mm margin, and the difference was significant for GI_high_, CI, and nCI (*p* < 0.001).

**Table 3:** Dosimetric parameters in comparison between CyberKnife and single-isocenter volumetric modulated arc therapy (SI-VMAT) with a 1- and a 3-mm PTV margin.

	**CyberKnife**	**SI-VMAT (3 mm)**	**SI-VMAT (1 mm)**	***p* (Wilcoxon SR)**	***p* (Wilcoxon SR)**
	**Median (Q1–Q3)**	**Median (Q1–Q3)**	**Median (Q1–Q3)**	**CK vs. SI-VMAT (1 mm)**	**SI-VMAT: 1 vs. 3 mm**
**Dose to the healthy brain**					
Mean dose (Gy)	0.109 (0.103–0.113)	0.376 (0.355–0.462)	0.375 (0.343–0.419)	**<0.001**	0.188
*V*_3GyPlan_ (ml)	75.7 (55.3–93.9)	819 (686.6–897.2)	802.1 (694.6–886.4)	**<0.001**	0.813
*V*_5GyPlan_ (ml)	24.3 (17–28.2)	384.5 (317.7–422.7)	383.9 (296.9–401)	**<0.001**	0.438
*V*_8GyPlan_ (ml)	10.6 (7.6–12.4)	99 (92.2–142.1)	89.9 (85.5–93.3)	**<0.001**	0.188
*V*_10GyPlan_ (ml)	7.1 (5–8.5)	54.3 (53.8–66.7)	47.8 (44.9–49.7)	**<0.001**	0.125
*V*_12GyPlan_ (ml)	4.9 (3.4–5.9)	33.7 (33.6–40.3)	29.4 (28.2–30.1)	**<0.001**	0.188
*V*_3GyTarget_ (ml)	11.7 (7.9–12.6)	99.7 (85.8–136.5)	102.4 (86.8–126.6)	**<0.001**	0.625
*V*_5GyTarget_ (ml)	3.5 (2.4–3.8)	48.1 (47–52.9)	49.5 (48–49.8)	**<0.001**	0.313
*V*_8GyTarget_ (ml)	1.6 (1.1–1.6)	15.4 (14.9–15.8)	13.3 (12.9–13.6)	**<0.001**	0.313
*V*_10GyTarget_ (ml)	1.1 (0.7–1.1)	9 (7.8–9.1)	7.1 (7.1–7.5)	**<0.001**	0.188
*V*_12GyTarget_ (ml)	0.7 (0.5–0.8)	5.4 (4.2–5.6)	4.3 (4.2–4.5)	**<0.001**	0.141
**Gradients and conformity**					
GI_high_	3.1 (3–3.1)	5.4 (4.8–5.4)	6.7 (5.2–6.9)	**<0.001**	**<0.001**
GI_low_	3.0 (3.0–3.0)	5.7 (5.3–6.7)	7 (6.6–7.8)	**<0.001**	0.283
CI	1.2 (1.2–1.2)	1.6 (1.6–1.8)	2.8 (2.7–2.9)	**<0.001**	**<0.001**
nCI	1.2 (1.2–1.3)	1.7 (1.7–1.9)	2.8 (2.7–2.9)	**<0.001**	**<0.001**

*Numbers in bold indicate statistical significance*.

*Wilcoxon SR, Wilcoxon signed-rank test; V_XGy_, volume of healthy brain receiving X Gy; GI_high_, gradient index for high-dose range; GI_low_, gradient index for low-dose range; CI, RTOG conformity index; nCI, new conformity index*.

## Discussion

We performed a systematic treatment plan comparison, evaluating conventionally planned SI-VMAT against the CyberKnife M6 dedicated radiosurgery system for the stereotactic treatment of 5–10 BM. The CyberKnife system outperformed SI-VMAT in all regarded dosimetric parameters, however at the cost of relevantly increased treatment time.

Several reports have been published recently, comparing commercially available automated single-isocenter VMAT solutions among each other or with the Gamma Knife, respectively (10–12, 25, 26). The reported findings vary considerably regarding different plan quality metrics. Those metrics consequently have to be discussed individually and in conjunction with respective differences in crucial baseline parameters such as the number and size of the treated lesions or hardware-related aspects such as MLC leaf width.

An important quality metric in cerebral SRS is represented in the HB volume receiving 12 Gy (*V*_12Gy_) (27–30). High volumes for *V*_12Gy_ and *V*_10Gy_ have been associated with a risk for necrosis of up to 10%. Flickinger et al. have developed a correlation model based on Gamma Knife treatment plans to predict the risk of symptomatic radionecrosis according to *V*_12Gy_ volume (31). This and similar models, derived from data for the SRS of arteriovenous malformations, set the recommended thresholds for *V*_12Gy_ at ~10 cc (31, 32). The *V*_12Gy_ volumes found when treating multiple targets with single-isocenter VMAT techniques are considerably higher than the 10 cc recommended for single-target treatments, although the impact on toxicity outcomes in this constellation is unclear: Ruggieri et al. and Potrebko et al. reported mean values for *V*_12Gy_ of 23–39 cc for HyperArc and Elements MBM, while Potrebko et al. found a *V*_12Gy_ of 24 cc for Gamma Knife (12, 25). Notably, the mean number of targets (five vs. eight lesions) differed between the aforementioned publications, as well as mean total target volume (9.6 vs. 1.16 cc). Furthermore, *V*_12Gy_ values are reported with confidence intervals amounting to up to 100%, indicating great variability between individual treatment plans. Ruggieri et al. thus suggested dividing the *V*_12Gy_ by the number of individual targets as a more reliable way of assessing high-dose falloff outside the individual target lesion and to better relate to the thresholds recommended in the literature (25). The median value of 6.2 cc per target we found for SI-VMAT following this approach compares favorably to those of 3–7 cc reported by Ruggieri et al. and Hofmaier et al. and falls well within the recommended threshold of 10 cc (11, 25).

Thomas et al. went even further by suggesting a linear model to quantify the correlation between *V*_12Gy_ and number of targets as well as total target size (10). It has to be noted that the median *V*_12Gy_ value of 6.5 cc (1.1 cc per target) we found for CyberKnife treatment plans was consistently lower than the model-based prediction, as well as the figures we found in our analysis or that are reported in the literature for either SI-VMAT or Gamma Knife (10, 12). However, they concur with values recently reported for CyberKnife by Kadoya et al. (26). This finding suggests a true superiority of CK-SRS in conformity for the treatment of multiple BMs. A possible explanation lies in the additional non-isocentric beam angles that are possible with CK-SRS, since the robot is able to position the treatment head at a greater variety of positions and directions from the source field. In combination with the CyberKnife's circularly collimated beam profile that favors sharp dose gradients, this could prove a sizeable advantage in conformity, especially for small peripheral lesions. Another important metric to assess dose conformity is the conformity index in its various established forms. As explained earlier, in this analysis, we primarily used the new conformity index (nCI), defined as the reciprocal of the modified Paddick conformity index and which, for SRS, typically yields values that are similar to or slightly larger than the RTOG CI (20–22). The median nCI we found for SI-VMAT at 1.6 is in agreement with the figures reported by Potrebko et al. (1.6–1.7 in the per-PTV analysis) (12). It is higher than that reported by Thomas et al. and Ruggieri et al., who found Paddick CI values in the vicinity of 0.7–0.9, corresponding to nCI values of 1.1–1.4 (10, 25). The most probable explanation here is the use of a high-definition MLC by Thomas et al., featuring a leaf width of 2.5 mm at the isocenter, while the agility MLC used in our study had double that leaf width at 5 mm. The median nCI value we found for CK-SRS at 1.2 was considerably lower than the one for SI-VMAT. Here again, we hypothesize the CyberKnife's abovementioned greater number of non-isocentric beam angles, combined with finer beam collimation to be the deciding factors for superior conformality. Our findings in this regard contradict those reported by Kadoya et al., who found CI values of 0.6 for CK-SRS, corresponding to an nCI of 1.7, which was significantly inferior to the reported figures for SI-VMAT (26).

To assess the impact of MLC leaf width on conformity and high-dose exposure of the healthy brain surrounding the targets, we compared our results—achieved with a 5-mm leaf width—to those analyses in the literature that expressly utilized an MLC with 2.5 mm leaf width for SI-VMAT (10, 12, 26). *V*_12Gy_ volumes in those publications ranged from ~1 cc (26) to 3–4 cc (10) per target. Notably, between those publications—all utilizing the same MLC and treating rather small total GTVs ( ≤ 1 cc)—the *V*_12Gy_ per target increased with the median number of BM per patient. An analogous trend was detectable for the conformity index, which also deteriorated with a rising number of targets. Our results for SI-VMAT, utilizing a 5-mm MLC (*V*_12Gy_ = 6.2 cc per target), are only slightly inferior to those discussed above, whereas our results for CyberKnife (*V*_12Gy_ = 1.1 cc per target) compare favorably despite the number of BM being ≥5 in our analysis. In summary, this suggests that a smaller MLC leaf width has an effect on overall SI-VMAT plan conformity, especially for treatment plans with a limited number of rather small BM. However, this effect becomes less pronounced with an increasing number of targets and total GTV size, as the target number and total size weigh more heavily then. This deduction concurs with the findings expressed in the linear correlation model suggested by Thomas et al. (10) and discussed above in more detail.

In our analysis, we chose different margin widths for SI-VMAT and CK-SRS, respectively. Our rationale in this approach is clinical necessity: Opinions regarding the merits and drawbacks of margins in cerebral SRS widely vary between institutions and are frequently discussed in the literature. However, conclusive analyses have shown that especially rotational positioning errors can cause significant underdosage in treatment plans for multiple brain metastases. This effect is aggravated with smaller target size and further distance from the isocenter (13, 14, 33). It is consequently warranted to compensate for those uncertainties with margins of 2–3 mm on treatment machines that lack continuous intrafractional motion correction, as is available on the CyberKnife. It was the aim of our analysis to realistically compare two treatment techniques, as they would be employed in clinical practice. Consequently, the choice of margin width had to be made according to the uncertainties dictated by the respective treatment machine and could not be identical for CyberKnife and SI-VMAT.

Our results for the separate comparison of SI-VMAT with a 1-mm PTV margin vs. a 3-mm PTV margin illustrated that the margin width does not relevantly impact the mid- and low-dose exposure of the surrounding healthy brain (brain—PTV) in the context of SI-VMAT. *V*_*X*__Gy_ values were not significantly different between SI-VMAT plans with a 1- vs. a 3-mm margin. However, conformity and gradient indices (=high-dose exposure) deteriorated with the use of a 1-mm PTV margin. For the current analysis, this can be explained with target size: Since the analysis included predominantly small BM (median metastasis size <0.1 cc), the reduction in PTV margin width to 1 mm results in PTV volumes too small for agility MLC with 5-mm leaf width to cover conformally. In the context of the current analysis, which assumes the unavailability of intrafractional motion compensation on the machine delivering SI-VMAT, this aspect is irrelevant, for a 3-mm margin is clinically required. For systems where the delivery of SI-VMAT with a 1-mm PTV margin is feasible with the help of intrafractional motion compensation and/or a hexapod couch, our results suggest that the use of a high-definition MLC with 2.5-mm leaf width would be recommendable when treating very small BM.

It is unclear whether the dosimetric advantages we found for CK-SRS over SI-VMAT regarding conformity and dose exposure of the healthy brain are clinically relevant. To the best of our knowledge, there are to date no systematic analyses examining the clinical implications of conformity differences within the range discussed above. On the other hand, treatment time for CK-SRS was significantly increased in comparison to SI-VMAT. Depending on the clinical constellation, number of targets and individual patient performance, treatment time can be relevant for deciding on the feasibility of radiosurgery. Pain, e.g., from bone metastases or respiratory insufficiency, is frequent in the patient collective presenting with multiple BM. Those and similar clinical factors can be decisive in limiting a patient's ability to tolerate prolonged radiosurgical treatment with mask-based head fixation. Patients thus unsuitable for radiosurgery are typically referred to WBRT—exposing them to increased toxicity—or best supportive care. SI-VMAT, with its decisively shorter treatment time and higher availability, may provide the means of offering those patients the benefits of radiosurgery while making some compromises in terms of dosimetric plan quality. The results of our analysis suggest that clinically acceptable SI-VMAT plans can be achieved without the requirement of a dedicated solution such as HyperArc or MBM, further increasing the availability of this treatment approach.

Our study has several limitations: RayStation-based treatment planning of SI-VMAT for multiple BM lacks several features available in dedicated systems for SI-VMAT such as HyperArc or MBM by Brainlab. Among those are automated algorithms for the optimization of couch and collimator angles, arc length, and weighting. Those features may contribute to overall plan quality, increasing high-dose falloff outside the target. A high-definition MLC and making use of leaf interdigitation to prevent interlesions dose bridges are additional factors that could further improve dose conformity and that were unavailable on the system used for our current analyses.

Our study is strengthened by its sample size, which is larger than comparable dosimetric analyses and allows for the results to be conclusively and significantly demonstrated. No comparable data exist for the role of conventionally planned SI-VMAT as an alternative to the CyberKnife system, specifically for the treatment of >5 BM. Our study is further distinguished by its use of margins adapted to the respective treatment technique, which is rarely done in comparable dosimetric studies, as discussed above. In consequence, the results of our analysis mirror realistic clinical scenarios and are directly applicable in clinical practice. Finally, although all treatment plans fulfilled the criteria for clinical acceptability, this is a dosimetric study. Clinical trials are warranted to evaluate if the dosimetric differences detected are relevant, e.g., lead to an increase in treatment-associated toxicity.

## Conclusion

The present analysis is the first to systematically compare SI-VMAT against the CyberKnife M6 system for the radiosurgical treatment of 5–10 BM. SI-VMAT offers enhanced treatment efficiency, as compared to CyberKnife, but requires compromise regarding conformity and integral dose to the healthy brain. Additionally, delivery at a conventional linac may require a larger PTV margin to account for delivery and setup errors when intrafractional motion compensation is unavailable. Further evaluations are warranted to determine whether the detected dosimetric differences are clinically relevant. SI-VMAT could be a reasonable alternative to a dedicated radiosurgery system for selected patients with multiple BM.

## Data Availability Statement

The datasets generated for this study will not be made publicly available since national legislation and the terms of study ethics approval do not allow dataset sharing outside of the institutions participating in the analysis.

## Ethics Statement

This study was performed following institutional guidelines and the Declaration of Helsinki of 1975 in its most recent version. Ethics approval for the study and a waiver of written informed consent were granted by the Heidelberg University ethics committee on April 12, 2018 (#S-172/2018). Patient confidentiality was maintained by anonymizing patient data to remove any identifying information.

## Author Contributions

RE, DB, JD, and SR planned and supervised this analysis as part of the neuro-radiooncological research group. ET-M and DS performed comparative treatment planning. AC performed data extraction and review. DW performed all statistical analysis. RE reviewed data analysis and drafted the manuscript. DS, KL, LK, SH, TF, BN, and SA contributed patient data and participated in reviewing and improving analysis and manuscript. All authors read and approved the final manuscript.

## Conflict of Interest

The authors declare that the research was conducted in the absence of any commercial or financial relationships that could be construed as a potential conflict of interest.
